# Patient perspectives on single-port versus multi-port robotic-assisted urologic oncology procedures: a survey-based analysis

**DOI:** 10.1007/s11701-026-03652-7

**Published:** 2026-07-20

**Authors:** Rahul Nalluri, Kashish Khanna, Ramya Chamkeri, Trushar Patel

**Affiliations:** 1 University of South Florida Health Morsani College of Medicine, Tampa, FL USA; 2 AdventHealth, Tampa, FL USA; 3 Tampa General Hospital, Tampa, FL USA

**Keywords:** Urologic oncology, Single-port, Multiport, Cosmesis, Patient preference, Robotic surgery

## Abstract

The da Vinci single-port (SP) system enables robotic urologic surgery through a single incision, unlike the conventional multiport (MP) approach. Although SP surgery may reduce postoperative pain and improve cosmesis, its adoption remains limited by a steep learning curve, high costs, and limited long-term oncologic data. This study evaluated patient perspectives on SP versus MP robotic-assisted urologic surgery, focusing on the relative importance of surgical and cosmetic outcomes. We conducted a cross-sectional survey of 51 adults scheduled for robotic-assisted genitourinary oncologic procedures in a single surgeon’s practice between 2024 and 2025. Participants completed a Likert-scale questionnaire assessing cosmetic outcomes, cancer control, postoperative pain, complication risk, cost, and marketing influence, and ranked five surgical factors by importance. Statistical analyses included Friedman rank testing, Wilcoxon signed-rank tests, Mann-Whitney U tests, and Kruskal-Wallis tests. Of 58 eligible patients approached, 51 completed the survey. Cancer control was ranked as the most important factor (mean rank 1.47 ± 0.81, *p* < 0.001), followed by complication risk and postoperative pain. Cost and cosmetic appearance were ranked least important. Cosmetic prioritization did not differ significantly across age, sex, or diagnosis groups. Male patients placed greater importance on cancer control than female patients (*p* = 0.002). Among men, those without prostate cancer placed greater importance on postoperative pain than those with prostate cancer (*p* = 0.006). Patients undergoing robotic genitourinary oncologic surgery prioritize cancer control, complication risk, and postoperative pain over cost and cosmetic outcomes when choosing between SP and MP robotic platforms.

## Introduction

The da Vinci single-port (SP) system was approved for urological surgery in 2018. Early data suggests that SP technology may reduce post-operative pain, shorten hospital stays, and improve cosmetic outcomes when compared to multiport surgery [1, 2].

Despite its potential advantages, single-port (SP) surgery remains a subject of ongoing debate when compared with multiport (MP) approaches, largely due to its steep learning curve, significant implementation costs, and the absence of robust long-term oncologic outcome data [3, 4]. The existing literature suggests that overall patient satisfaction appears comparable between single-port (SP) and multiport (MP) approaches. However, patients undergoing SP surgery tend to report greater satisfaction with cosmetic outcomes, particularly scar appearance [5]. Despite this, current evidence does not clearly define how much weight cosmetic benefits carry in relation to other clinical outcomes when patients make surgical decisions [5, 6].

Patient preference for cosmetic outcomes is influenced by demographic and clinical context. Younger patients tend to place greater importance on cosmesis, often favoring smaller or concealed incisions and showing more willingness to accept higher risk or cost for improved appearance [7–10].

Surgical indication also shapes how much patients value cosmetic outcomes. Those undergoing benign procedures tend to place greater importance on appearance, while patients having cancer surgery prioritize treatment effectiveness and avoiding complications, with cosmesis being less important. There is no strong cross-specialty evidence demonstrating that cosmetic preferences differ meaningfully among specific oncologic procedures [7, 8].

This study seeks to address this knowledge gap by evaluating the relative importance of attributes associated with single-port (SP) versus multiport (MP) robotic surgery from the patient perspective. We specifically examine the role of cosmetic outcomes and hypothesize that scar appearance has a limited influence on decision-making in genitourinary (GU) oncologic procedures.

## Methods

We conducted a cross-sectional survey study using a Likert-scale questionnaire to understand patient perspectives regarding SP vs. MP robotic surgery at our institution. This study was designed and reported in accordance with STROBE recommendations for cross-sectional studies. It was designed to be a 5-minute survey to minimize responder fatigue and maximize the accuracy of the responses. Fifty-one consecutive, consenting patients were surveyed over a six-month period (2024–2025) from a single surgeon’s practice. Eligible participants were adults (> 18 years) scheduled for robot-assisted surgery for renal, bladder, or prostate tumors, undergoing their first planned robot-assisted genitourinary oncologic procedure.

**Fig. 1:**
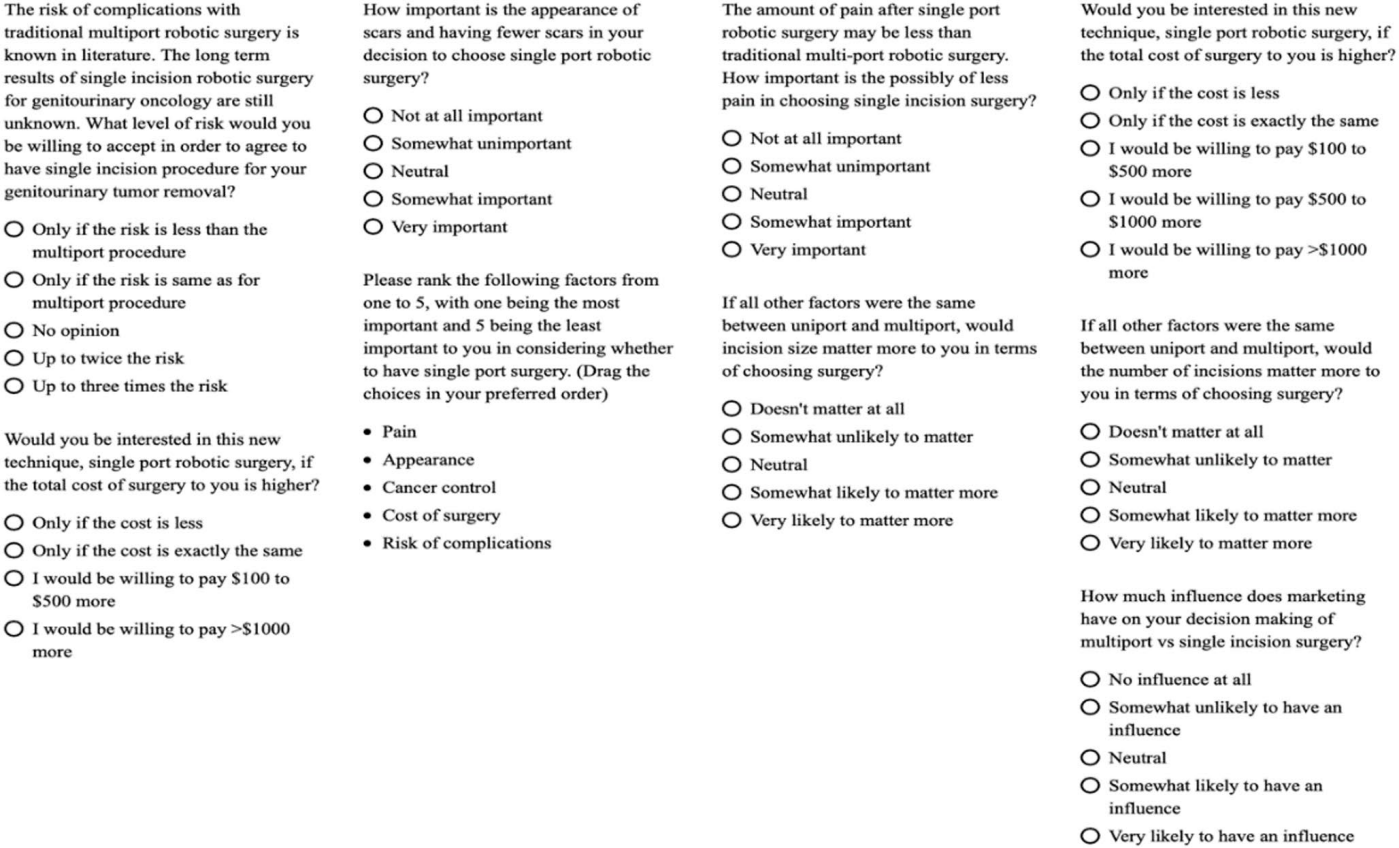
Survey Instrument

The primary outcome was patient preference regarding factors influencing the choice between SP and MP robotic surgery. Domains assessed included cosmetic outcomes, oncologic efficacy, marketing, cost, postoperative pain, and complication risk (survey instrument shown in Fig. 1). Six items were measured using a 5-point Likert scale, and one item required respondents to rank surgical attributes by importance.

Key predictors included age range, sex, and tumor type. Responses were collected and managed via Qualtrics. All demographic variables were self-reported, and surveys were completed in-person, in a single sitting, without external assistance.

Likert-scale responses were analyzed as ordinal data, with descriptive statistics reported as medians, interquartile ranges (IQR), and response frequencies. Ranked response questions were analyzed using Friedman mean rank test and pairwise comparisons were assessed using Wilcoxon signed-rank tests which utilized a Bonferroni-adjusted significance level. Categorical group differences were assessed using the Mann–Whitney U test and Kruskal–Wallis test. All analyses were performed using IBM SPSS v31.

## Results

58 eligible patients were approached and 51 consented and completed the survey without attrition. Demographics are summarized in (Table 1).

**Table 1:** Patient Demographic, Clinical, and Social Characteristics

Characteristic	*N* = 51
**Age Range (in years)** 18–50 51–70 71+	4 (8%) 32 (63%) 15 (29%)
**Sex** Male Female	40 (78%) 11 (22%)
**Race/Ethnicity** Caucasian Black/African American Hispanic/Latino	29 (56%) 12 (24%) 10 (20%)
**Diagnosis/Type of Tumor** Prostate Kidney Bladder Adrenal	26 (51%) 21 (41%) 3 (6%) 1 (2%)
**Prior Abdominal Surgery** Yes No	33 (65%) 18 (35%)

### Likert-scale

**Fig. 2:**
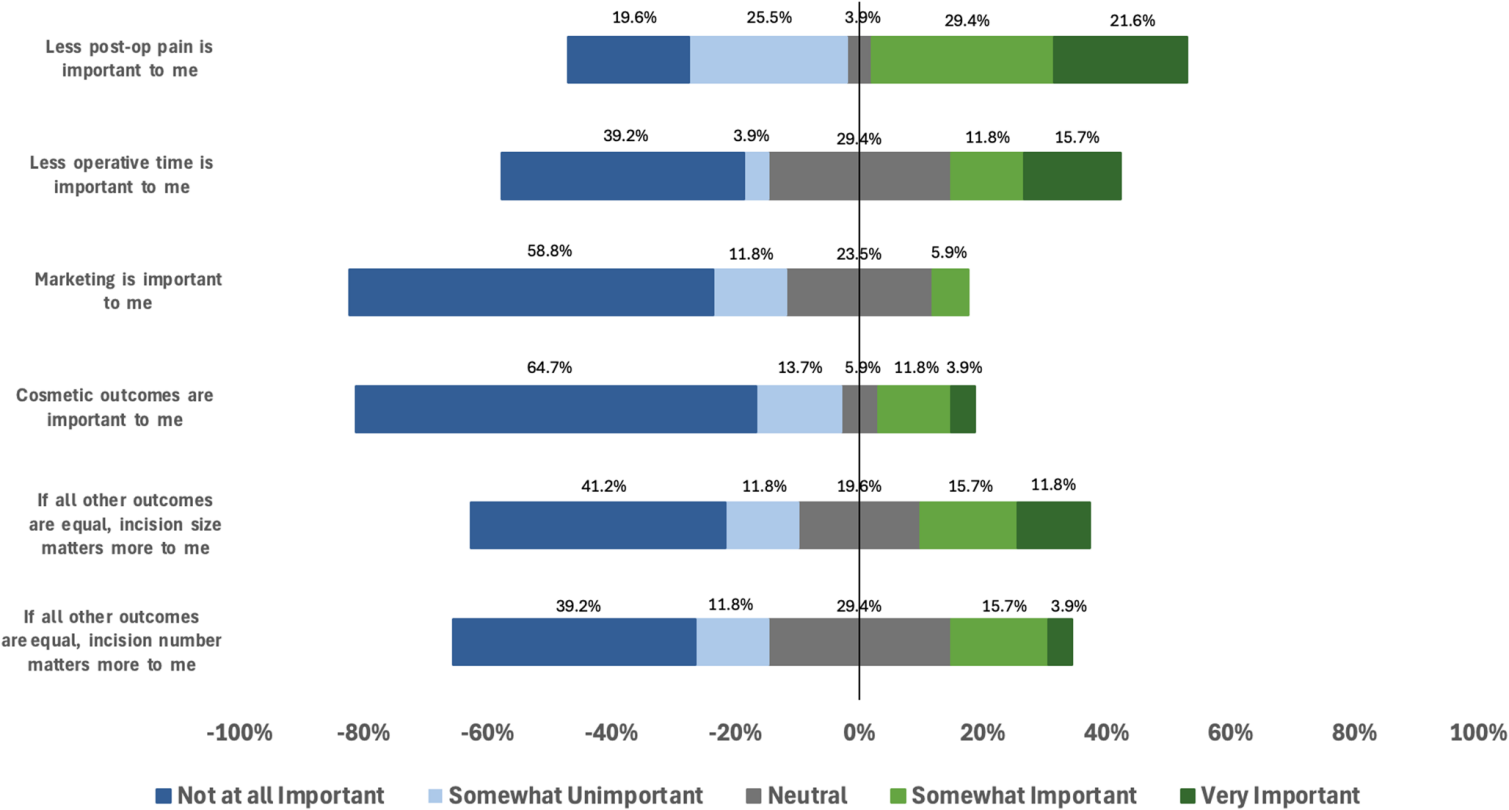
Patient Perspectives on Attributes for Choosing Between SP and MP Robotic Surgery

Patient perspectives on SP vs. MP robotic surgery were assessed using Likert-scale questions with a focus on different surgical characteristics (Fig. 2). There were no significant differences in responses to Likert-scale questions by sex are age group.

### Ranking data

**Fig. 3:**
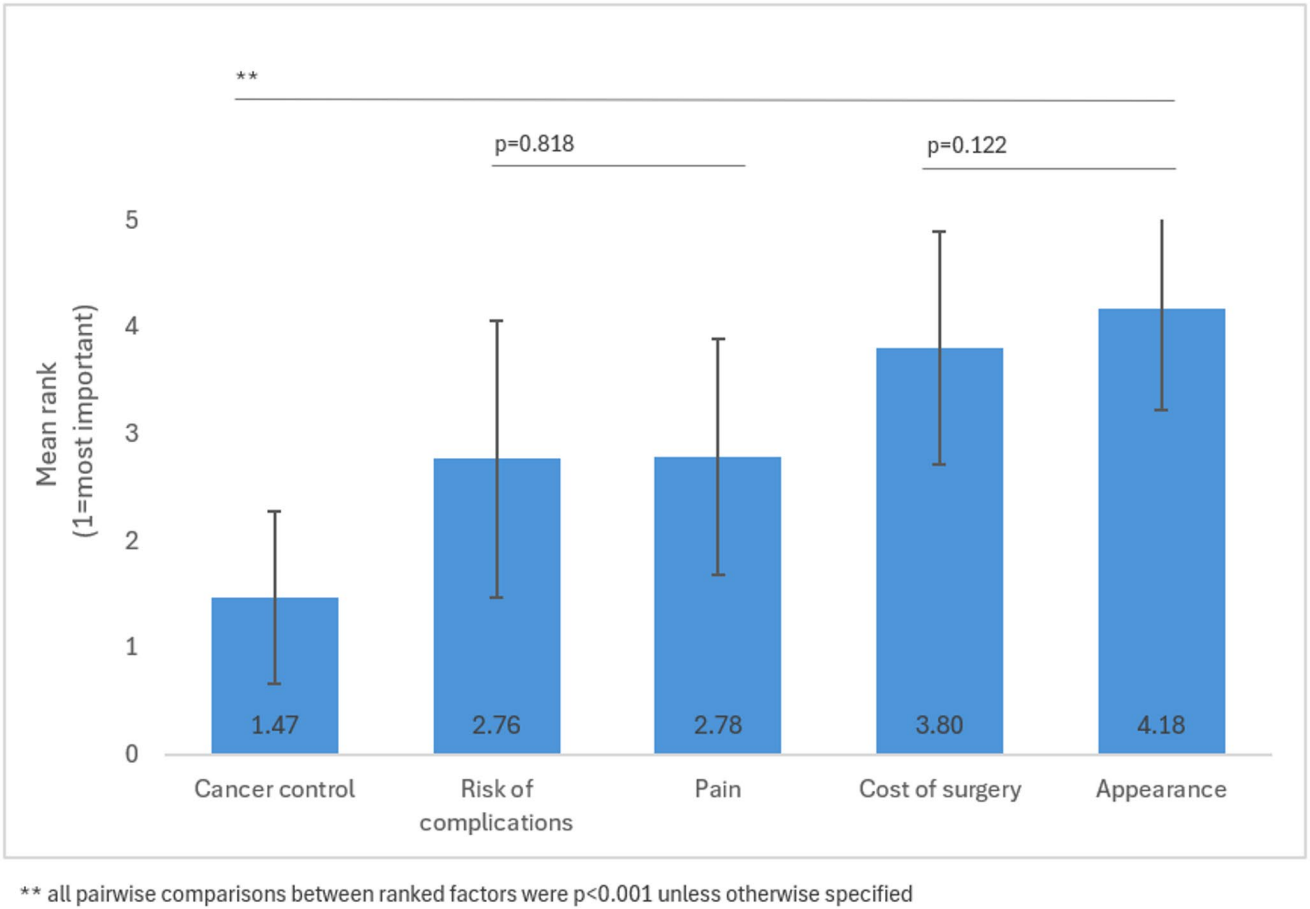
Surgical Outcomes Ranking with Frequency of Rank. Error bars represent standard deviation. ** all pairwise comparisons between ranked factors were *p* < 0.001 unless otherwise specified

Participants were asked to rank five factors in terms of importance, including pain, appearance, cancer control, cost, and risk of complications. Overall, the Friedman test showed a significant difference between factors (*p* < 0.001). Cancer control was ranked as most important, with mean rank 1.47 ± 0.81, significantly more important than any other factor (*p* < 0.001 for all pairwise comparisons). Risk of complications and postoperative pain were ranked second and third, prioritized over cost and scar appearance (*p* < 0.001 for both comparisons) with no difference between pain and complication rankings (*p* = 0.818). Cost and scar appearance were ranked lowest overall (*p* < 0.001), with no significant difference between them (*p* = 0.122) (Fig. 3).

As shown in Table 2, cancer control was the highest priority and scar appearance the lowest across all age groups. When stratified by sex, males placed significantly greater emphasis on cancer control compared to females (1.28 ± 0.60 vs. 2.18 ± 1.08, *p* = 0.002). Females, in contrast, assigned relatively greater importance to complication risk; however, this difference did not remain statistically significant after adjustment for multiple comparisons (*p* = 0.026). Across both prostate and non-prostate cancer groups, scar appearance was consistently ranked as the least important factor. Because all patients with prostate tumors were male, we performed a Mann–Whitney U test comparing males in the prostate tumor group to males in the non-prostate tumor group to control for sex as a confounder. There was no significant difference in the ranking of cancer control between groups; however, postoperative pain was assigned greater importance by males in the non-prostate cancer group compared to those in the prostate cancer group (*p* = 0.006).

**Table 2:** Subgroup analyses of ranked surgical outcomes by sex, diagnosis and prior abdominal surgery, assessed using Mann-Whitney U tests

Surgical Outcome	Age group				Sex			Diagnosis			Prior Abdominal Surgery		
	≤ 50 *N* = 4	51–70 *N* = 32	71+ *N* = 15	*p*-value	Male *N* = 40	Female *N* = 11	*p*-value	Prostate *N* = 26	Other *N* = 25	*p*-value	Yes *N* = 18	No *N* = 33	*p*-value
Pain	3.25 ± 1.26	2.47 ± 1.02	3.33 ± 1.05	0.035*	2.68 ± 1.10	3.18 ± 1.08	0.154	3.00 ± 0.98	2.54 ± 1.22	0.106	2.94 ± 1.16	2.70 ± 1.08	0.347
Appearance	4.00 ± 0.82	4.25 ± 0.95	4.07 ± 1.03	0.652	4.10 ± 0.96	4.45 ± 0.93	0.204	4.12 ± 1.03	4.25 ± 0.90	0.714	4.50 ± 0.79	4.00 ± 1.00	0.068
Cancer control	1.50 ± 1.0	1.47 ± 0.8	1.47 ± 0.83	0.986	1.28 ± 0.60	2.18 ± 1.08	0.002**	1.15 ± 0.46	1.83 ± 0.96	0.003**	1.83 ± 0.99	1.27 ± 0.63	0.017*
Cost of surgery	3.75 ± 1.26	3.81 ± 1.06	3.8 ± 1.21	0.993	4.00 ± 0.85	3.09 ± 1.58	0.095	3.96 ± 0.87	3.58 ± 1.28	0.459	3.44 ± 1.42	4.00 ± 0.83	0.226
Risk of complications	2.5 ± 1.73	3.00 ± 1.27	2.33 ± 1.18	0.111	2.95 ± 1.28	2.09 ± 1.14	0.026*	2.77 ± 1.24	2.79 ± 1.38	0.934	2.28 ± 1.02	3.03 ± 1.36	0.049

*significant at *p* < 0.05 significance level

**significant at *p* < 0.01 adjusted significance level

## Discussion

We evaluated the da Vinci single-port (SP) system and the da Vinci multi-port (MP) system from the patient perspective to determine which surgical attributes most strongly influence decision-making. Within our cohort, cancer control was the highest-ranked factor influencing surgical decision-making between these modalities [1–4]. However, patient priorities may differ according to disease state, treatment context, and the outcomes being considered, including functional recovery, quality of life, continence, and sexual function, particularly among patients with prostate cancer.

While the finding that patients prioritize cancer control and complication risk over cosmetic outcomes may appear intuitive, its relevance within the SP versus MP robotic surgery debate has not been well characterized. As institutions increasingly adopt SP technology and surgeons continue to navigate the associated learning curve, understanding which platform-specific attributes patients value most becomes increasingly important. Our findings suggest that patient decision-making is driven primarily by oncologic efficacy and safety rather than cosmetic considerations, providing a patient-centred perspective that complements the predominantly surgeon- and outcome-focused literature. These data may help guide patient counselling and support shared decision-making as newer robotic technologies continue to evolve.

Contrary to the emphasis often placed on surgical innovation, most patients disagreed that cosmetic outcomes including incision size, number, and appearance, were important factors in selecting the SP platform over MP. The median Likert score for all cosmetic outcomes was 1 (“strongly disagree”), with 50–79% of respondents dismissing its importance. These results align with broader surgical literature suggesting that while SP may improve isolated scar satisfaction, patients undergoing abdominal oncologic procedures do not view cosmesis as a primary driver [11, 12, 16–19]. Similarly, external factors like marketing and operative time received median scores of 1, suggesting they have negligible influence on patient preference.

51% of participants considered postoperative pain an important factor in choosing a surgical platform, while 45% did not, highlighting variability in patient preferences. Patients aged 51–70 assigned significantly less importance to postoperative pain compared to other age groups. This finding aligns with prior literature, which suggests that younger patients tend to experience and prioritize postoperative pain more than older individuals. While cancer control was the top priority for all, the non-prostate group (nephrectomy and cystectomy) placed significantly higher importance on postoperative pain. This likely reflects the more invasive nature and higher recovery demands of these procedures, suggesting the SP platform’s potential pain-reduction benefits may be most valued in these specific populations [13–15].

Operative time has been reported to be comparable or slightly longer with the SP robotic platform. However, increased operative duration likely reflects the early learning curve rather than intrinsic limitations of the technology and may normalize as surgeons gain experience with the SP system. It is reasonable to infer that operative duration is not a primary factor influencing patient preference when choosing between the two approaches.

To summarize, although cosmetic outcomes were consistently ranked as low priority, younger patients and patients without prostate cancer assigned greater importance to postoperative pain. These findings suggest that patient groups expected to place greater value on recovery-related outcomes may be more receptive to potential perioperative advantages associated with the SP platform. Future studies should further explore whether these populations derive greater perceived benefit from SP surgery.

This study has several limitations that should be considered when interpreting the findings. The primary limitation is the relatively small sample size and the uneven distribution of participants across age, sex, and diagnostic categories, which may limit generalizability and subgroup analysis should be viewed with caution.

Additionally, the survey instrument was developed by the study team to evaluate factors considered clinically relevant to SP versus MP surgical decision-making. Although designed to minimize respondent burden and maximize completion rates, the instrument was not formally validated prior to administration. Hence the survey instrument was institution-specific, not externally validated, which may limit reproducibility. In addition, diagnostic subgroup analysis was constrained by the need to group non-prostate malignancies together due to limited numbers, and interpretation of diagnosis-based differences is further complicated by sex confounding, as all prostate cancer patients were male. Notably, the initially observed higher prioritization of cancer control in the prostate cancer group was no longer significant after adjusting for sex. As such, these results should be viewed as preliminary and hypothesis-generating. We also acknowledge that the ultimate procedure that these patients underwent or were scheduled to undergo after counselling was not recorded at the time of taking this initial survey as this may have influenced their perspective and act as a selection bias. Of note, these surveys were administered during preoperative clinic visits before discussion of the planned procedure but were not used to guide surgical platform selection. Surgical decision-making remained at the discretion of the treating surgeon and standard clinical practice. Patient responses may sometimes be influenced by pre-existing treatment expectations and counselling received before surgery or elsewhere at encounters with another Urologist prior to our survey. Previous studies have demonstrated that patients who have selected or been assigned a treatment strategy may perceive that option more favorably despite uncertainty regarding comparative outcomes [20]. Consequently, some survey responses may reflect choice-supportive perceptions rather than entirely independent preferences. This possibility should be considered when interpreting the findings.

Although previous studies have demonstrated that patients undergoing cancer surgery generally prioritize treatment efficacy and safety, few studies have specifically evaluated how patients weigh these considerations in the context of SP versus MP robotic platforms. The present study extends existing literature by quantifying the relative importance of oncologic outcomes, complications, pain, cosmesis, and cost within a contemporary genitourinary oncology population. These findings may help inform patient counselling and provide context for the ongoing adoption of SP technology.

This study was conducted during a period of increasing adoption of the single-port platform in urologic surgery and was designed to provide insight into patient perspectives in the context of the ongoing SP versus MP debate. Understanding these preferences offers a complementary lens to traditional perioperative and oncologic outcomes and may help inform future, larger-scale investigations.

## Conclusion

In this cohort of patients undergoing robotic genitourinary oncologic surgery, cancer control and complication risk were prioritized above cosmetic outcomes including incision size and number when choosing between single-port (SP) and multi-port (MP) robotic surgical platforms. Cosmetic factors appeared to have limited influence on decision-making, whereas postoperative pain remained important for selected patient groups including younger age and non-prostate cancer urological oncology groups. These findings may help inform patient counselling and provide insight into patients who may place greater value on the potential perioperative advantages associated with SP surgery.

## Data Availability

The patient surveys that support the findings of this study are available from Qualtrics, but restrictions apply to the availability of these data, which were used under license for the current study and so are not publicly available. The data are, however, available upon request.
